# Cardiac imaging highlights from European Society of Cardiology 2024: the future is within our grasp!

**DOI:** 10.1093/ehjimp/qyaf009

**Published:** 2025-02-05

**Authors:** Stefano Figliozzi, Erika Hutt, Alessia Gimelli, Wael A Jaber

**Affiliations:** IRCCS Humanitas Research Hospital, Via Alessandro Manzoni 56, Rozzano, Milan 20089, Italy; Department of Biomedical Sciences, Humanitas University, Via Rita Levi Montalcini 4, Pieve Emanuele, Milan 20072, Italy; Department of Cardiology, Mid America Heart Institute, Saint Luke’s Health System, Kansas City, MO 64111, USA; Imaging Department, Fondazione Toscana Gabriele Monasterio, Pisa, Italy; Heart and Vascular Institute, Cleveland Clinic Foundation, Cleveland, OH, USA

**Keywords:** multimodality imaging, advances, ESC scientific meeting, outcomes

## Abstract

The European Society of Cardiology has held its annual Congress in London, UK, from 30 August to 2 September 2024. With a total of 31 800 participants, 5400 faculty and presenters, and many National Cardiac Societies and industry partners, the Congress has taken an enormous step forward to present and discuss the latest advances in cardiovascular medicine. The sizable intercontinental reach was proved by the fact that 5 of the 10 top countries, in terms of submission of abstracts, were from outside Europe: China, the USA, Japan, Korea, and Australia. This brought a great impetus for international collaboration and exchange of views, learning from different perspectives. Specifically, the field of cardiovascular imaging has been in the spotlight, remarking its growing, central, and transversal role in modern cardiovascular medicine. In this communication, we offer a summary of some notable advances in research, either in terms of novelty or clinical applicability, within the realm of four imaging modalities: echocardiography, cardiovascular magnetic resonance, computed tomography, and nuclear imaging.

## Highlights in echocardiography

### Machine learning applied to colour-Doppler videos to assess the severity of heart valve regurgitation

Echocardiographic assessment of valvular heart regurgitation is a complex task requiring multi-parametric assessment, often leading to uncertainties and discrepancies in defining the severity of aortic regurgitation (AR), mitral regurgitation (MR), or tricuspid regurgitation (TR), which can greatly impact on the decision to intervention, follow-up frequency, and cardiovascular outcomes. Artificial intelligence (AI) offers promising solutions to reduce these uncertainties and discrepancies, enhancing the reliability of echocardiographic assessment.

From Columbia University Irving Medical Centre, New York, USA, Long *et al*. proposed a deep learning system for AR, MR, and TR quantification. A large number of transthoracic echocardiograms (TTE) were split into train (*n* = 65 301), validation (14 018), and test sets (13 746). The Deep Learning for Echo Analysis, Tracking, and Evaluation of Valvular Regurgitation (DELINEATERegurgitation) system identified colour Doppler clips and yielded a classification of AR, MR, and TR on a six-grade scale (none/trace, mild, mild-moderate, moderate, moderate-severe, and severe) using the ‘human cardiologist’ interpretation as a reference standard. The system used a hybrid neural network, leveraging the spatiotemporal feature learning capabilities of convolutional neural networks with the sequential processing strengths of transformer networks. In the validation model, the DELINEATERegurgitation system showed a high accuracy in the classification of AR (*k* = 0.857), MR (*k* = 0.867), and TR (*k* = 0.847), predicting the same severity classification as cardiologists (AR 91.8%, MR 79.9%, and TR 77.7%) or within ±1 grade accuracy (AR 99.5%, MR 98.6%, and TR 98.6%).^1^

Thus, the proposed DELINEATERegurgitation AI system demonstrated high accuracy in the quantification of AR, MR, and TR using colour Doppler TTE videos, paving the way for future works focusing on automating quantification of AR, MR, and TR for clinical implementation and decision-making. The future is to validate these findings across different machine vendors and in a multicentre registry.

### Prognostic value of left atrial strain in mitral stenosis

Mitral stenosis causes an inflow restriction during diastole, rendering conventional echocardiographic algorithms for assessing diastolic dysfunction invalid, and alternative parameters are needed to estimate the effects of increased left atrial (LA) pressures and their clinical implications.

Using 2D strain imaging, Chan *et al*.,^2^ representing a study group from Malaysia and Singapore, aimed to assess the prognostic value of LA strain in 237 adult patients with moderate to severe mitral stenosis. The study endpoint was a composite outcome of all-cause mortality, heart failure hospitalization, and valve intervention (i.e. percutaneous transvenous mitral commissurotomy and surgical intervention). The median follow-up was 4.54 years (interquartile range [IQR] 1.44–6.44). Among several clinical and echocardiographic parameters, LA reservoir strain with a cutoff <15.8% was independently associated with the study endpoint on multivariable Cox regression analysis [hazard ratio (HR) 2.51, 95% confidence interval (CI) 1.54–4.10], whereas LA conduit strain, contractile strain, and volume index did not demonstrate any significant association with the endpoint. This result is of potential immediate clinical applicability in this challenging cohort of patients. The results went beyond describing an echocardiographic abnormality in the LA strain and linked the abnormality to increased mortality.

## Highlights in cardiovascular magnetic resonance

### Clinical implications of late gadolinium enhancement granularity in patients with hypertrophic cardiomyopathy

The presence of late gadolinium enhancement (LGE) has been associated with adverse clinical outcomes in patients with hypertrophic cardiomyopathy (HCM). ‘Zooming in on the images’, the study group directed by Dr Theo Pezel, Université Paris Cité, aimed to assess the incremental prognostic value of the granularity of LGE, including extent, location, and pattern in patients with HCM to predict all-cause death.^3^

**Figure 1:**
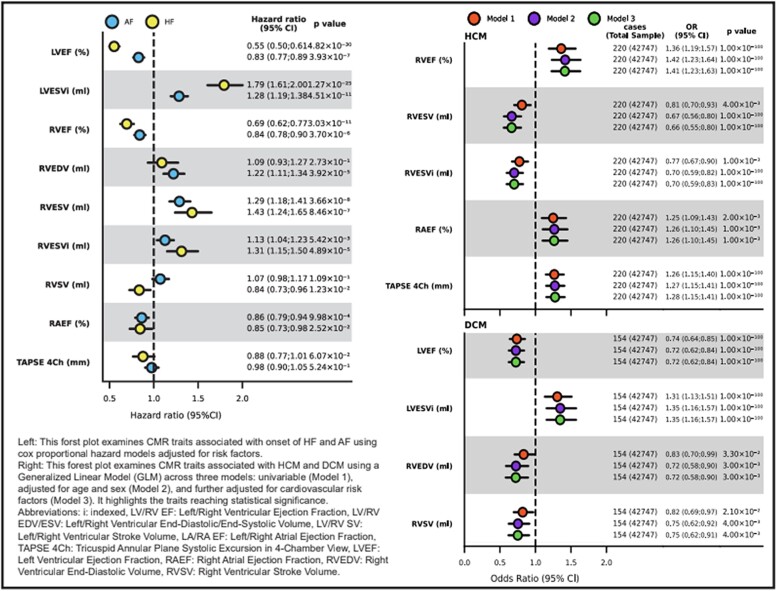
Forrest plot describing CMR traits associated with HF and AF (left panel) and HCM and DCM (right panel). Courtesy of Dr P.M. Croon.

**Figure 2:**
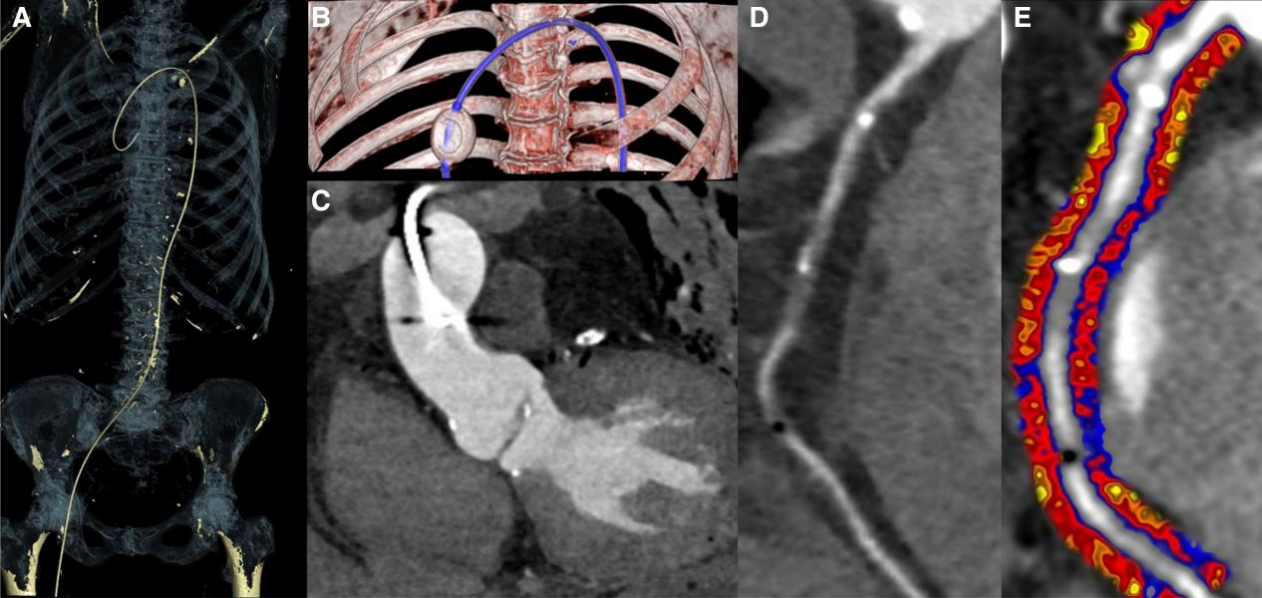
Example of post-mortem volume rendered CCTA (*A*) demonstrating balloon occlusion angiography at the aortic root (*B*, *C*) with resulting coronary imaging (*D*) and post-processed perivascular fat inflammation map (*E*). Courtesy of Dr C. Xie.

Among 2672 HCM patients (52 ± 7 years, 56% males), 862 (32%) had LGE. After a median (IQR) follow-up of 9^4–8^ years, 447 (17%) patients died. After adjustment for traditional prognosticators, the presence of LGE was strongly associated with all-cause death (adjusted HR 3.96, 95% CI: 3.26–4.80, *P* < 0.001). In the subgroup of patients with LGE (*n* = 862), survival curves showed that all parameters defining the LGE granularity were associated with a higher risk of all-cause death (all *P* < 0.001, *Figure 1*). A nested Cox model adjusted on traditional prognosticators showed that the LGE extent, location, and pattern were all independently associated with all-cause death (all *P* < 0.001, *Figure 2*). The model of ‘LGE granularity’ combining all independently significant LGE features showed the best improvement in model discrimination and reclassification above traditional prognosticators (C statistic improvement: 0.90; net reclassification index = 41.9%; integrated discrimination improvement = 13.2%, χ^2^ global = 450, all *P* < 0.001; LRtest *P* < 0.001, *Figure 2*). Based on a large group of patients, these findings encourage the assessment of ‘LGE granularity’ to improve the risk stratification of patients with HCM. It would be interesting to test this granularity of LGE uptake within the current ESC HOCM risk calculator.

### AI to unveil CMR traits linked to cardiomyopathy mutations in phenotype naive carriers

In genetic mutation, carriers of HCM and dilated cardiomyopathy (DCM), disease onset is highly variable, and some people do not experience any disease at all. Croon *et al*.^9^ from the University of Amsterdam, the Netherlands, aimed to identify CMR biomarkers of HCM and DCM in phenotype naive individuals. CMR and whole genome sequencing (WGS) (*n* = 42 723) data were sourced from a UK Biobank sample free from heart failure (HF) at the time of CMR measurements. A validated deep learning network derived 22 CMR traits. Using WGS data, the authors identified 154 people with an HCM mutation and 220 with a DCM mutation, where HCM carriership was associated with five and DCM with four CMR traits. These included right atrial ejection fraction (EF) [odds ratio (OR) 1.26; 95% CI 1.10–1.45], and right ventricular-EF (OR 1.41: 95% CI 1.23–1.63) for HCM, and LV-EF (OR 0.72; 95% CI 0.62–0.84), and right ventricular stroke volume (OR 0.75; 95% CI 0.62–0.91) for DCM. By combining deep learning of CMR with a large sample size WGS, the authors were able to identify CMR biomarkers of HCM and DCM carriership in apparently healthy subjects. This could prove relevant for early identification and monitoring of phenotype naive people. In the coming years, it is exciting to see how the genetic high-risk markers and the identified CMR markers will perform in a synergistic/complementary fashion to identify the high risk for sudden death or worsening NYHA class.

### Persistence of LA prothrombotic flows after aortic valve replacement for aortic stenosis

Severe aortic stenosis (AS) is known to be associated with ischaemic stroke independently of atrial fibrillation (AF), and the risk for stroke remains high even after successful aortic valve replacement (AVR). To better understand the underlying pathophysiology, a study group directed by Saul Meyerson, University of Oxford, Oxford, UK, assessed whether severe AS leads to prothrombotic LA flow changes with reduction of LA velocities and vorticity, which might not normalize after AVR.^10^

The authors included 40 adult patients with severe AS without AF (age 73 ± 10 years, CHA2DS2VASc = 3) and 37 controls (age 71 ± 6 years, CHA2DS2VASc = 3). All participants underwent CMR including the assessment of LA 4D flow. Out of the 40 patients with severe AS, 23 (58%) underwent a successful AVR and repeated CMR scan 6 months after surgery. When compared with matched controls, patients with severe AS had greater LV mass, smaller LA and LV volumes, and reduced global longitudinal strain (GLS) (all *P* < 0.05). The LA EF did not differ among groups, but severe AS was associated with slightly lower LA peak flow velocity (mean 0.25 vs. 0.28 m/s, respectively) and lower LA flow vorticity (mean 16.6 vs. 21.1 radians) compared with controls (both *P* < 0.05). Successful AVR led to a reduction of LV mass, but post-AVR patients displayed persistently smaller LA and LV volumes, and persistently reduced GLS compared with controls (all *P* < 0.05), as well as significantly lower LA peak flow velocity (mean 0.24 vs. 0.28 m/s, *P* = 0.003) and vorticity (17.1 vs. 21.1 radians, *P* < 0.001). Thus, patients with severe AS displayed prothrombotic LA flow characteristics persisting after successful AVR despite normalization of LV hypertrophy. From this small sample, future studies should focus on the detection of permanent and potentially irreversible LA dysfunction in patients with severe AS and link these changes to strokes and the development of AF. These early findings open the way for novel research fields in 4D flow CMR, potentially improving the risk stratification for stroke independent of AF and CHADs-Vasc score in patients with AS.

## Highlights in cardiac CT

### Coronary inflammation in patients without obstructive coronary artery disease

Investigators from the Oxford risk factor and non-invasive imaging (ORFAN) study evaluated the prognostic value of coronary inflammation using the fat attenuation index (FAI) score and an AI-integrated prediction model in patients without obstructive coronary artery disease (CAD) on coronary CT angiography (CCTA). This study included 40 091 patients from seven UK hospitals and a median follow-up of 2.7 years as well as a nested cohort of 3393 patients with 7.7 years of median follow-up.^11^

This group found that (i) patients with non-obstructive CAD accounted for 63.7% of all cardiac deaths highlighting the importance of better risk stratification tools in this patient population; (ii) FAI score in any of the three epicardial coronaries was significantly associated with cardiac mortality with or without obstructive CAD; (iii) the highest quartile of FAI score in the left anterior descending artery showed 20-times higher risk of cardiac death; and (iv) the AI-integrated risk model using coronary inflammation, plaque burden and clinical risk factors demonstrated good alignment with observed events in the whole population and reclassified patient’s risk above clinical risk factor-based prediction.

Thus, this new technology, which has been in development for nearly a decade continues to demonstrate its value and we expect its incorporation in clinical practice and possibly future guidelines for the purpose of individual risk stratification of patients who undergo CCTA.

### Post-mortem coronary inflammation

This fascinating study was performed by the Oxford Group again using the Caristo FAI software but this time the question was somewhat unusual.^4^

They sought to understand if the FAI score and the AI-Risk (FAI score + plaque characteristics + clinical risk factors) obtained by post-mortem CCTA could help adjudicate coronary vs. non-coronary deaths as determined by autopsy.

Thirty-seven adults with sudden death of unknown cause were included. Aortic root angiogram with aortic occlusion balloon was followed by CT acquisition. Two experienced radiologists reported the cause of death with and without the aid of the FAI score and AI-Risk results.

Using a threshold of 42% for AI-Risk and 34 for FAI score, 18 true positives, 2 false positives, and all true negatives were detected. Both radiologists identified 17/18 coronary deaths, 5 false positives, and 8 false positives, respectively. With the aid of the FAI score, the radiologists’ concordance was 100%. Thus, the authors concluded that this minimally invasive approach of post-mortem CCTA has the potential of increasing diagnostic accuracy of the cause of death. The cause of sudden death and attribution to different disease entities has implications for blood relatives, disease management and treatment, and socioeconomic allocation of scarce health care resources. This kind of work gets us closer to understanding the ‘final frontier’.

### SGLT2 inhibitors and coronary plaque burden

Zhang *et al.* from Beijing, China investigated whether SGLT2 inhibitors affect coronary plaque among T2DM patients with angina pectoris who underwent serial CCTA. Patients who underwent CCTA within 3 months prior and 6 months after initiation of SGLT2 inhibitor were included and matched 1:1 with similar patients who did not initiate SGLT2 inhibitor.^5^

They found that in patients taking SGLT2 inhibitors, the volume of non-calcified and low attenuation plaque decreased between the two examinations while it did not among propensity-matched patients who were not taking SGLT2 inhibitors.

**Figure 3:**
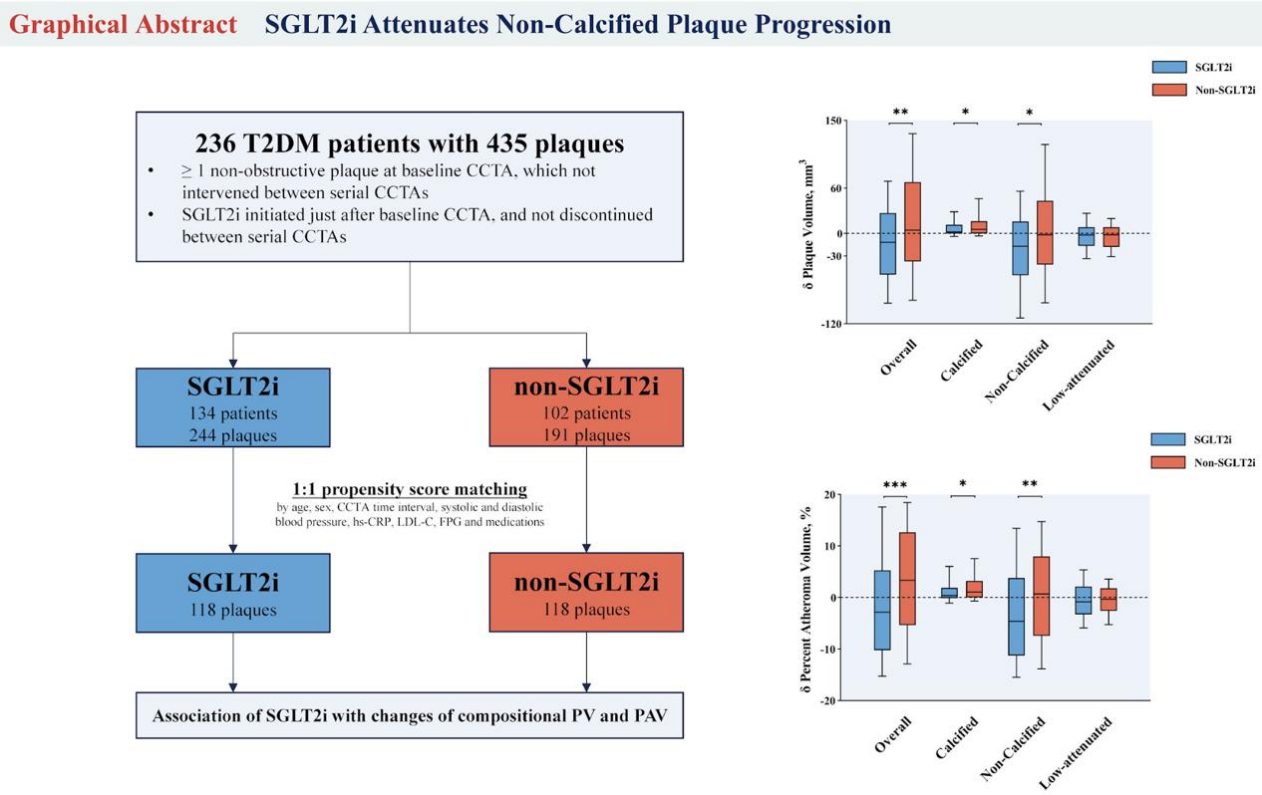
Flow diagram and results of plaque volume and atheroma volume on CCTA based on SGLT2 use status. Courtesy of T. Zhang.

Overall, this suggests that SGLT2 inhibitors may regress coronary plaque and explain some of the cardio-protective effects of these medications in contemporary clinical trials. As the use of SGLT2 inhibitors expands and their impact on risk reduction beyond diabetic patients is reported, plaque volume assessment can help us to understand how these medications work in addition to blood sugar control, and reduction in blood pressure (*Figure 3*).

### A novel morphological classification of LA appendage with potential clinical impact

**Figure 4:**
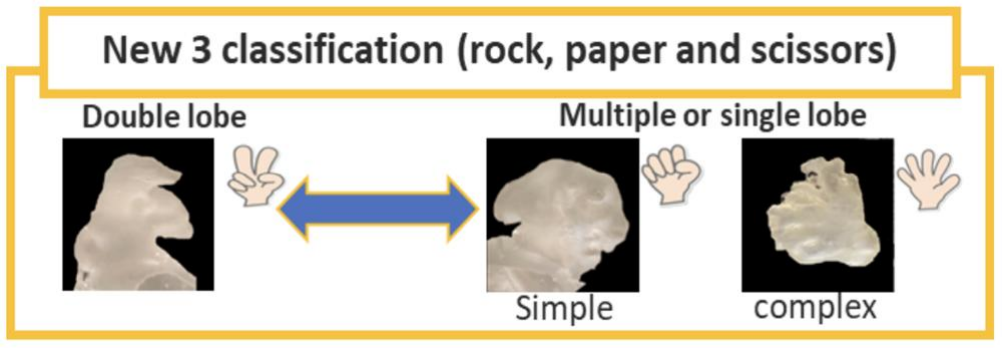
Pictorial of new LAA morphology classification with clinical impact. Courtesy of Dr S. Ryuzaki.

The clinical usefulness of currently adopted left atrial appendage (LAA) morphological classifications remains questionable. A study group from Chiba University Graduate School of Medicine, Japan, aimed to identify the relationship between a novel and simple approach to morphological LAA characterization and the risk of embolic events. Three-dimensional printing of 94 LAAs was generated from cardiovascular CT imaging data. Thirty (26%) LAA shapes were classified as ‘scissors’ (i.e. bi-lobe), 38 (33%) as ‘paper’ (i.e. complicated peripheral branches), and 48 (41%) as ‘rock’ (i.e. simple). The three groups of patients were matched for comorbidities, and 51 (44%) patients had a history of cardioembolic stroke (18% vs. 30% vs. 53%; Rock vs. Scissors vs. Paper, *P*-value = 0.05). In the multivariate analysis, the Paper morphology type was associated with the risk of cardioembolic stroke (*P*-value = 0.01). In addition, of those, 36 (31%), 32 (28%), 28 (24%), and 20 (17%) patients were divided into the conventional classification of Chicken wing, Cactus, Cauliflower, and Windsock types, respectively. The four types were not associated with cardioembolic stroke (18% vs. 25% vs. 55%; Chicken wing vs. Cactus vs. Cauliflower vs. Windsock types, *P*-value = 0.83). Larger studies are needed to confirm whether this novel and simple morphological approach to LAA could have clinical implications for the prevention of embolic risks related to LAA thrombi. As the use of CT to assess the LAA before and after pulmonary vein isolation and LAA closure expand, this simple classification may enhance our ability to select the patients at the highest risk for stroke to treat aggressively and potentially early LAA closure (*Figure 4*).

## Highlights in nuclear cardiac imaging

### Imaging post-infarct inflammation

Investigators from the UK evaluated the role of somatostatin receptor subtype 2 (SST2) positron-emission-tomography/magnetic-resonance-imaging (PET/MRI) using ^68^GaGa-DOTATATE after myocardial infarction (MI). This was a prospective observational cohort study of 38 participants with recent MI (58% ST-elevation-MI) who underwent ^68^GaGa DOTATATE PET at 2 weeks and 3 months post-MI and compared MRI characteristics and volumetric changes at 1 year, as well as circulating immune cell phenotyping and serum proteomic markers.^7^

The first finding was that ^68^GaGa DOTATATE maximum standardized uptake value (SUVmax) was elevated in areas of MI and correlated well with regional wall motion abnormalities, LGE and T_1_ times by MRI. Secondly, at 3 months, the mean infarct max decreased, and this again correlated well with reduction in T_2_-weighted oedema signal and serum biomarkers of cardiac injury and systemic inflammation. Thirdly, residual inflammation at 3 months correlated with change in indexed LV end-diastolic even after adjusting for LV infarct size and clinical variables. Fourthly, the 3-month to 2-week ratio of mean infarct SUVmax was the strongest predictor of adverse myocardial remodelling and the CCL25, a chemokine involved in macrophage recruitment, was the biomarker most associated with higher 3-month to 2-week ratio.

**Figure 5:**
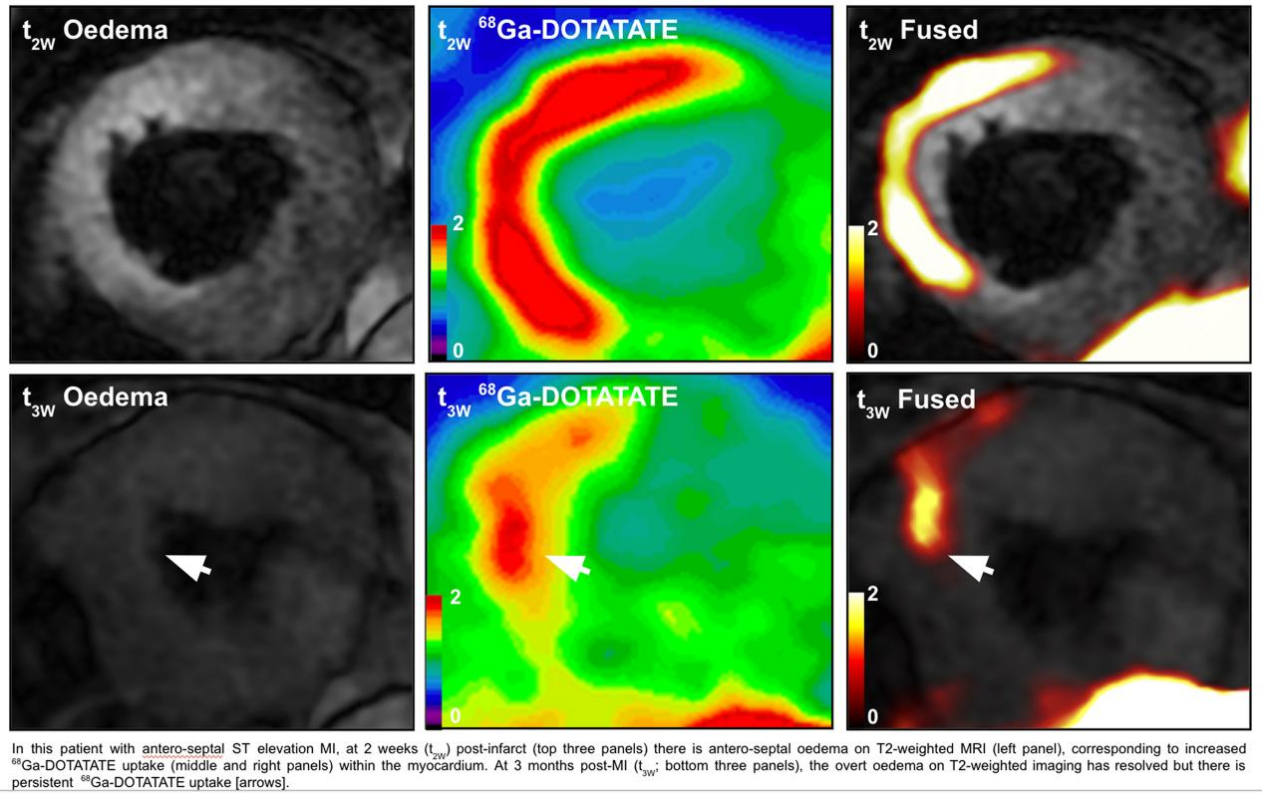
Example of post-MI imaging after 2 weeks (upper panel) and 3 months (lower panel) of the event in a single patient demonstrating resolution of oedema but persistence of ^68^Ga-DOTATATE. Courtesy of A. Corovic.

These findings are promising and shed light on the role of imaging inflammation after infarct, which can potentially guide future immunotherapy as well as viability assessment. We may be able in the future, using these data, to understand why different patients with similar size infarct undergo different paths for LV remodelling and the development of heart failure (*Figure 5*).

### INOCA diagnosis using ^13^N-ammonia PET

Sakai *et al*. identified 98 patients with non-obstructive CAD who underwent NH3-PET for evaluation of INOCA. They applied a feature-tracking algorithm to calculate circumferential strain at rest and with stress. Global myocardial perfusion reserve (MFR) and myocardial strain ratio (MSR-stress/rest strain) were obtained. Among 98 patients, 47 were diagnosed with INOCA based on ESC guidelines. MSR and MFR were significantly lower in patients with INOCA compared with those without INOCA. Survival rates were lower in patients with INOCA and low MSR.^8^

**Figure 6:**
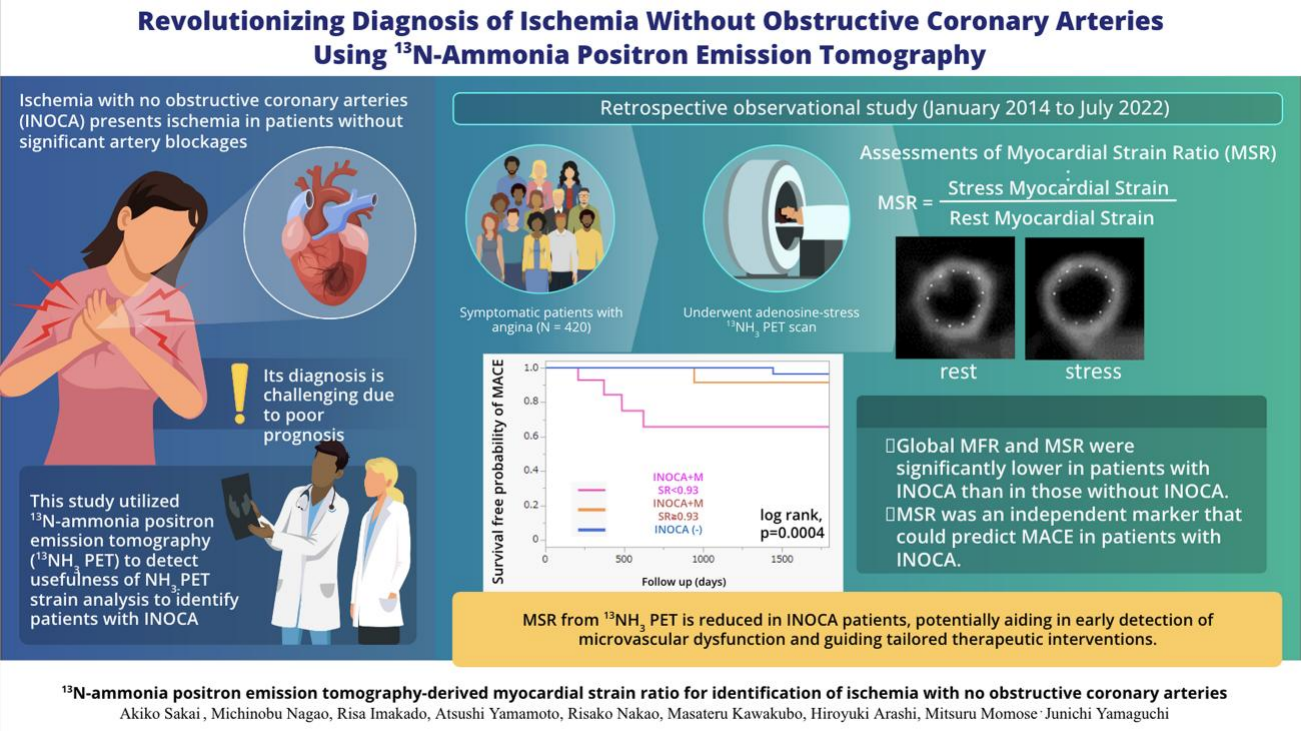
Graphical abstract of retrospective observational study evaluating the role of MSR and MFR in INOCA. Courtesy of A. Sakai.

This study shows us that strain by NH3-PET may help identify and predict outcomes of patients with INOCA (*Figure 6*).

### Sex differences in coronary plaque activity with 18^18^F-NaF PET CT

Authors from Edinburgh compared plaque characteristics of a large cohort of patients (*n* = 999) with coronary atherosclerosis who had undergone ^18^F-NaF PET CT angiography. Women only comprised 15% (*n* = 151) of the cohort.^12^ They found that women had lower coronary calcium scores and coronary microcalcification activity (CMA). However, after adjusting for clinical comorbidities and coronary calcium scores, there was no sex-related difference in CMA values. Finally, while CMA was associated with MI in both men and women, the increase in hazard ratio was not different between sexes.

The authors conclude that while men have higher coronary calcification, there is no difference in plaque activity between men and women and the latter is a stronger predictor of future MI regardless of sex. This may identify CMA as a ‘sex-blind’ independent cardiovascular risk factor. Future studies with a larger women cohort are necessary to determine the sex differences in coronary plaque activity with ^18^F-NaF PET CT. Our understanding of the contribution of CAD beyond the visual degree of stenosis has evolved over the years. Plaque anatomic characteristics, soft vs. calcified, and lipid core richness are now understood to contribute more to coronary events and sudden plaque rupture than visual lumen reduction. This very important study adds the missing 3D of plaque activity (hotness) to the predictive model of plaque rupture and may help us develop a ‘heat map’ beyond coronary artery calcification.

## Data Availability

No new data were generated or analysed in support of this research.

## Lead author biography

Dr Erika Hutt was born and raised in Costa Rica, where she studied Medicine at Universidad de Costa Rica. Her interest in clinical research led her to pursue medical training in the USA. Dr Hutt moved to the USA for Internal Medicine residency in 2016, after matching at the Cleveland Clinic Foundation. There she met her current husband and the couple matched into cardiology fellowship at CCF. She completed her general cardiology fellowship in 2022 as well as a Master’s degree in science in clinical research at Case Western Reserve University in 2023. In 2024, she completed a 2-year fellowship in Advanced Multimodality Cardiac Imaging at Cleveland Clinic Foundation and moved to Kansas City to start her practice at Saint Luke's Mid-America Heart Institute. Her current areas of interest are valvular heart disease, infiltrative cardiomyopathies, and multimodality cardiac imaging. During her free time, she enjoys cycling, hiking, and spending time with friends and family.
